# Effects of Smartphone Activities on Postural Balance in Adolescents with Intellectual Disabilities

**DOI:** 10.3390/children10111810

**Published:** 2023-11-14

**Authors:** Ghada Jouira, Cristina Ioana Alexe, Julien Narcis Herlo, Cristina Elena Moraru, Mihaela Bogdan, Dan Iulian Alexe, Gabriel Mareș, Sonia Sahli

**Affiliations:** 1 Research Laboratory Education, Motricité, Sport et Santé (EM2S) LR19JS01, High Institute of Sport and Physical Education of Sfax, University of Sfax, Sfax 3000, Tunisia; jouiraghada0825@gmail.com (G.J.); sonia.sahli@isseps.usf.tn (S.S.); 2 Department of Physical Education and Sports Performance, “Vasile Alecsandri” University of Bacău, 600115 Bacău, Romania; alexe.cristina@ub.ro; 3 Department of Physical Education and Sports Performance, “Aurel Vlaicu“ University of Arad, 310032 Arad, Romania; 4 Department of Physical Education and Sports, “Alexandru Ioan Cuza“ University of Iasi, 700506 Iasi, Romania; cristina.moraru@uaic.ro; 5 Doctoral School in Sport Science and Physical Education, “Alexandru Ioan Cuza“ University of Iasi, 700506 Iasi, Romania; mihaela.nicu@student.uaic.ro; 6 Department of Physical and Occupational Therapy, “Vasile Alecsandri” University of Bacău, 600115 Bacău, Romania; mares.gabriel@ub.ro

**Keywords:** adolescent, intellectual disability, center of pressure, timed up-and-go test, smartphone activities

## Abstract

Considering the rising prevalence of smartphone usage among adolescents with intellectual disabilities and their frequent motor challenges, understanding its impact on their physical well-being is important. This study aims to investigate the impact of smartphone activities on postural balance in adolescents with intellectual disabilities. Two groups of adolescents participated in the study: an intellectual disability group (IDG) (*n* = 16) and atypical development group (TDG) (*n* = 12). Static postural balance, using a stabilometric platform on firm and foam surfaces, and dynamic balance, using the Timed Up-and-Go Test (TUGT), were performed under various conditions, such as playing a game, watching videos, video calls, and listening to music. The center of pressure (CoP) values significantly increased (*p* < 0.05) during all smartphone activities (except listening to music) compared to the control condition in both groups, with the IDG demonstrated a more pronounced increase (*p* < 0.05) during playing video games and video calls on the firm surface. TUGT scores significantly increased (*p* < 0.05) during smartphone activities, with greater changes observed in the IDG (*p* < 0.05), and significantly decreased (*p* < 0.01) during listening to music in both groups. Our study suggests that adolescents with intellectual disabilities need special tools and guidance to ensure their safety and well-being when using smartphones.

## 1. Introduction

Adolescents with intellectual disabilities frequently exhibit diminished postural balance, as indicated by a consistent body of research [1,2,3,4,5], which can be attributed to deficits in visual perception, proprioceptive awareness, and vestibular inputs [3]. These deficits significantly impact overall mobility, making it challenging to perform movements and maintain postural balance [3]. Maintaining postural balance is crucial for daily life activities, and it involves the integration of sensory information from various systems in the body, including the visual, proprioceptive, and vestibular systems. However, when adolescents engage in multiple tasks simultaneously, a phenomenon known as dual-tasking postural control [6], their postural balance can be compromised due to divided attention. Previous research has explored dual-task models, particularly in the context of aging and neurodegenerative diseases, shedding light on the intricate interplay between cognitive and motor functions [7]. Furthermore, several studies have demonstrated that individuals with intellectual disabilities, including children and adults, experience alterations in postural balance when simultaneously engaging in supplementary tasks, affecting both static [6,8] and dynamic [9,10,11] balance compared to their counterparts with typical development. However, a noticeable research gap remains concerning the specific exploration of dual-tasking in adolescents with intellectual disabilities.

The rapid rise in smartphone usage, accelerated by the COVID-19 pandemic, has also affected adolescents with intellectual disabilities [12,13]. It has been previously demonstrated that adolescents with intellectual disabilities often participate in various smartphone activities, including playing games, watching videos, listening to music, and engaging in video calls [12]. These activities demand attention, cognitive involvement, and visual concentration, which can potentially influence the postural control system; however, the specific effects of such smartphone activities on the postural balance of adolescents with intellectual disabilities have not been explored yet. In individuals with typical development, research in this area has demonstrated that smartphone use can negatively affect postural balance. For instance, several studies have shown that engaging in smartphone activities, such as texting, browsing, watching videos, etc., lead to postural instability and compromised balance performance [14,15,16,17,18,19].

While the adoption of smartphones among adolescents with intellectual disabilities has provided potential benefits, including enhanced communication, education, and social engagement [12,13], it has also raised concerns about its impact on their physical well-being. Current studies on this topic are limited and inconclusive, making it necessary to conduct comprehensive research to assess the potential risks and benefits associated with smartphone use among this population. Given the motor impairments and cognitive challenges frequently experienced by adolescents with intellectual disabilities, it is essential to investigate the effects of smartphone activities on their postural balance.

Hence, the aim of this study is to investigate the effects of smartphone activities on postural balance in adolescents with intellectual disabilities. By exploring these effects, we aim to gain a better understanding of the challenges faced by this population and develop targeted interventions to optimize balance performance, reduce the risk of falls, and improve overall quality of life. We hypothesize that engaging in smartphone activities, such as playing games, watching videos, listening to music, and engaging in video calls would significantly affect postural balance in adolescents with intellectual disabilities, and that this effect varies depending on the specific activities involved.

## 2. Materials and Methods

### 2.1. Participants

**Figure 1:**
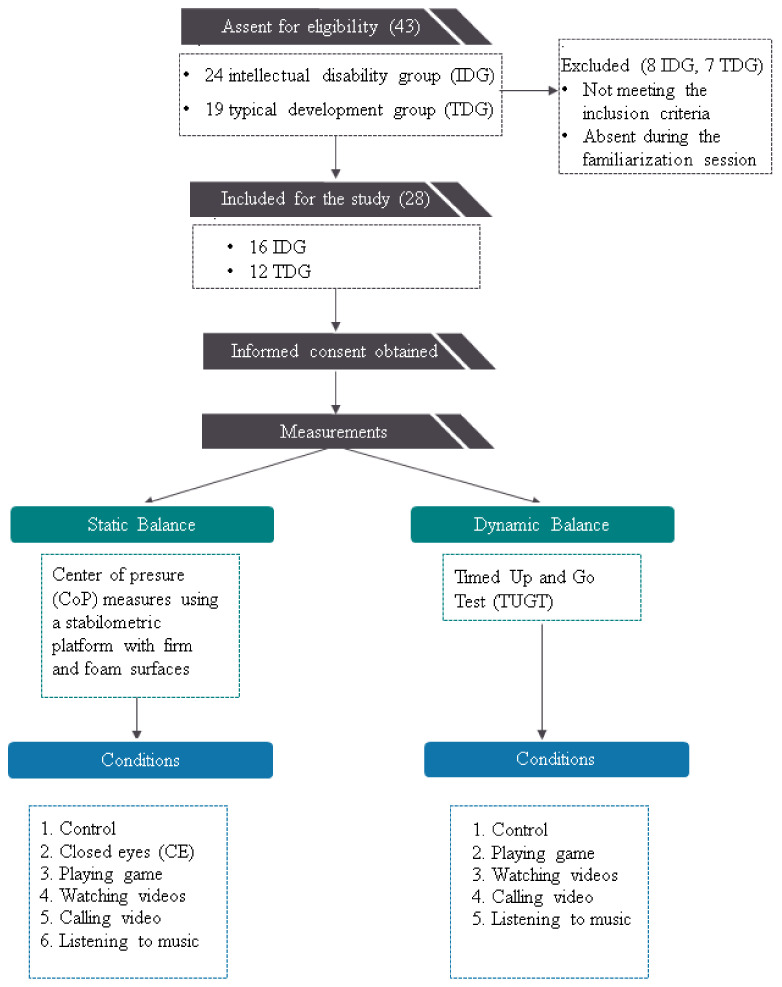
Flow diagram of sample selection and study design.

The sample size was a priori calculated using the software G*power for Windows (version 3.1.9.2; Heinrich Heine University Düsseldorf, North Rhine-Westphalia, Germany). Values for alpha, power, correlation among repeated measures, and non-sphericity correction (ε) were set at 0.05, 0.80, 0.50, and 1, respectively. To reach the desired power, data from at least 12 participants were deemed to be sufficient to minimize the risk of Type II statistical error. To accommodate the possible withdrawal of some participants, we recruited more participants than the number indicated by G*power (Figure 1).

The present study specifically targeted adolescents with intellectual disabilities who were enrolled in a specialized educational center. To ensure ethical compliance, formal permissions were obtained from the center authorities before initiating data collection. To recruit the sample, a three-stage screening process was employed. In the first stage, 24 adolescents with moderate to mild intellectual disabilities, as determined by the educational center psychologist using the Wechsler Intelligence Scale for Children—Fourth Edition (WISC-IV) [20], were randomly selected. For the typical development group (TDG), 19 adolescents without intellectual disability were selected from the nearest secondary school, matching the intellectual disability group (IDG) in terms of age, height, and weight (Table 1). In the second stage, 18 IDG participants and 14 TDG participants who met the inclusion and exclusion criteria were selected from the screened participants. The common inclusion criteria for both groups were: similar ethnicity, similar social class, low physical activity level (International Physical Activity Questionnaire score < 600 MET), not using neuroleptic medications or other substances that could affect postural balance, no history of lower limb injuries or surgeries within the past year, no visual and/or vestibular disorders. For the IDG, participants had to not use assistive devices for upright stance and walking. In the end, two individuals from each group were excluded from the study, as they were absent during the familiarization session, and the final sample comprised 16 individuals in the IDG and 12 individuals in the TDG (Figure 1).

Prior to initiating the study, a detailed explanation of the experimental protocol was delivered to the participants, parents, and caregivers involved. Written informed consent was obtained from the parents, and assent was sought from the adolescent participants. The assent process involved explaining the study’s purpose, procedures, and potential risks in a developmentally appropriate manner to ensure that the adolescents understood the study and were willing to participate. This study was conducted according to the Declaration of Helsinki and was approved by the Ethics Committee of the Vasile Alecsandri University of Bacău Romania (40/2/24 October 2023).

### 2.2. Study Design

This study employed a crossover comparative design to assess the effect of smartphone activities on static and dynamic balance in IDG and TDG participants. Static postural balance was measured using a stabilometric platform with firm and foam surfaces, while dynamic balance was evaluated using the Timed Up-and-Go Test (TUGT) (Figure 1). These measures were taken in various conditions, such as closed eyes (CE) (only in static postural balance), playing a game, watching videos, calling video, and listening to music. Consistency was maintained across experimental conditions for all participants. The study involved three laboratory visits separated by at least 2 days, with the first focusing on familiarization and the second and third involving the testing sessions in which we investigated the effects of smartphone activities on postural balance.

### 2.3. Measurements

#### 2.3.1. Static Postural Balance

In this study, participants were instructed to maintain a stable bipedal stance, barefoot, on a static stabilometric platform (PostureWin©, Techno Concept^®^, Cereste, France). This platform is recognized in the field of postural balance assessments for its high sampling frequency (40 Hz) and precise data acquisition (12-bits A/D conversion), which are essential for capturing subtle postural changes and fluctuations. It has been utilized in numerous postural studies, which adds to its validity and reliability [6,21,22].

During the experiment, participants stood on the platform under two different surface conditions. The first condition involved a firm surface, which was the rigid surface of the force platform itself. The second condition involved a foam surface, which consisted of a foam block (measuring 466 mm in length, 467 mm in width, and 134 mm in height above the ground). The foam block had a density of 21.3 kg/m^3^ and an elastic modulus of 20,900 N/m^2^. The foam surface was mounted on top of the rigid surface of the force platform. The use of this foam surface is based on prior studies that have demonstrated its effectiveness in challenging postural balance by introducing sensory perturbations [21].

The center of pressure (CoP) sways of each participant were recorded in a counter-balanced order in bipedal stance in each surface (firm and foam, respectively) under six different conditions, including:

- Control
- Closed eyes (CE)
- Playing game
- Watching videos
- Calling video
- Listening to music

All these conditions were designed and conducted to examine the effects of different surface conditions and sensory factors on the participants’ postural balance.

During the control condition, participants were instructed to maintain their gaze fixed on a 3 cm diameter target placed on the wall 3 m away from them in their horizontal field of view, which was chosen to establish a stable and controlled visual reference. In the CE condition, participants wore a blindfold to eliminate visual input and its contribution to postural balance. The inclusion of these conditions was to investigate the impact of visual input elimination on postural control, a well-documented aspect of static balance research [22]. Concerning the other conditions, participants were carefully positioned in the same stance to ensure consistency and eliminate the potential effects of altered postures. They were instructed to maintain this position as closely as possible throughout the experiment [14]. This approach aimed to minimize variations in posture, such as neck flexion or head tilting, which are commonly observed when individuals use their smartphones for activities such as talking or watching content or playing. Three trials were conducted for each experimental condition. Each trial lasted 30 s. To avoid potential effects of fatigue and learning, a 30 s rest period was provided between trials, during which participants were allowed to sit down if needed before returning to the platform (with the evaluator verifying their correct position). The parameter that is often used in static postural evaluations is the mean velocity of the CoP (CoP_Vm_). The CoP_Vm_ parameter is the sum of the scalar displacements of the CoP divided by the total time of the recording, expressed in mm/s. The CoP_Vm_ reflects the efficiency of the postural control system and characterizes the net neuromuscular activity required to maintain balance [23]. A smaller velocity is indicative of better postural control, and it is considered the most reliable measurement among trials [24].

#### 2.3.2. Dynamic Balance

Dynamic balance was evaluated using the Timed Up-and-Go Test (TUGT). This test is a well-established and recommended tool for evaluating dynamic balance and functional mobility, particularly in individuals with intellectual disabilities [25,26]. Participants were instructed to sit in a chair with their backs touching the backrest. When given the “go” signal, they had to stand up, walk a distance of 3 m at their normal walking speed (without running), turn around, and return to the chair. They then sat back down. The test was performed three times and the best time was recorded for analysis [27].

This test was assessed in five different conditions in a counter-balanced order as follows:

- Control
- Playing game
- Watching videos
- Calling video
- Listening to music

### 2.4. Interventions

#### 2.4.1. Playing Game

For the playing game condition, we conducted interviews with parents and caregivers of participants in the IDG to gather information about their child’s favorite game. The majority of participants mentioned puzzle games as their preferred choice, while a few expressed interest in other types of games, although they were still capable of playing puzzle games [28]. Based on this feedback and the caregivers’ recommendation, we made the decision to include a puzzle game, named “Pet Rescue Saga”, in the experimental protocol. The puzzle game consisted of a simple concept where the objective was to match three or more blocks of the same color together in order to remove them from the game board.

#### 2.4.2. Watching Videos

For the watching videos condition, we conducted interviews with the parents of the participants and discovered that they spent an average of 3 to 4 h daily watching videos on social media platforms such as TikTok and/or Instagram. All participants were either regular users with active accounts on these platforms or utilized their parents’ accounts for accessing them. Furthermore, both parents and participants were asked about their preferences regarding TikTok and/or Instagram videos. Based on the interview, the majority of participants had a particular interest in videos featuring cats and pets. Indeed, previous research showed that watching animal videos have several benefit on the psychological health [29,30] which has been associated with postural balance [31]. Throughout the experimental protocol, participants were instructed to watch videos specifically focused on cats and pets. To ensure consistency and minimize potential confounding factors, the same set of videos was used for all participants during the testing phase. This standardized approach aimed to reduce variations in video content and isolate the effects of the watching videos condition on postural balance.

#### 2.4.3. Video Call

Previous study showed that individuals with intellectual disabilities can use video call through social platforms [12,32]. In our study’s video call condition, participants engaged in a simulated conversation with a member of the research staff over the phone. The interview covered various topics, including personal information such as name, age, names of parents or siblings, hobbies, favorite foods, and preferred places. Questions like “What is your insert topic here?” were asked to prompt responses from the participants. These questions were adapted from previous research conducted in general population [14]. We modified and tailored these questions to suit our specific population. The answers provided by the participants during the interview were not recorded, as they were deemed irrelevant to the specific objectives of the study.

#### 2.4.4. Listening to Music

Regarding the listening to music condition, we conducted interviews with participants and/or parents and/or caregivers to determine their favorite songs. It was revealed that they enjoyed listening to pop music with fast tempo. Therefore, in the experimental protocol, participants were instructed to listen to pop music using headphones during postural balance tests. In fact, several studies in typical development individuals indicated that listening to music, regardless of the type and tempo, can have a positive effect on exercise behavior and/or perception [33,34]. To control for potential confounding factors and ensure consistency, the same set of music tracks was used for all participants.

### 2.5. Statistical Analysis

Data were analyzed using the program SPSS 25.0 (Statistical Package for the Social Sciences Inc., Chicago, IL, USA). The Kolmogorov–Smirnov test was used to verify the normality of the data distribution.

A three-way (6 Condition × 2 Group × 2 Surface) ANOVA with repeated measures was used to determine the effect of Condition (control/CE/playing game/watching video/video call/listening to music) and/or Group (IDG/TDG) and/or Surface (Firm/Foam) on the CoP_Vm_ values. In addition, a two-way (5 Condition × 2 Group) ANOVA with repeated measures was used to determine the effect of Condition (control/CE/playing game/watching video/video call/listening to music) and/or Group (IDG/TDG) on TUGT scores. To evaluate the changes in CoP_Vm_ values and TUGT scores after each condition, Δ CoP_Vm_% and TUGT% were calculated using the formula: Δ CoP_Vm_% or TUGT% = 100 × (condition − control)/condition. Subsequently, another two-way ANOVA with repeated measures was carried out to examine the effects of Δ changes in Condition and Group on CoP_Vm_ values (control/CE; control/playing game; control/watching video; control/video call; control/listening to music) and TUGT scores (control/playing game; control/watching video; control/video call; control/listening to music). A 95% confidence interval (CI) was calculated for means and Δ changes.

To determine whether the statistically significant differences found were practically significant, the effect size of each outcome measure was calculated. The partial eta squared (ηp^2^) formula was calculated for the main effects and interactions (small: 0.01 < ηp^2^ < 0.06; moderate: 0.06 < ηp^2^ < 0.14; large: ηp^2^ > 0.14), and the Cohen’s d was calculated for the pairwise differences (trivial: d < 0.2; small: 0.2 ≤ d < 0.5; moderate: 0.5 ≤ d < 0.8; large: d ≤ 0.8) [35]. To account for multiple comparisons, a Bonferroni adjustment was conducted. The level of statistical significance was set at *p* < 0.05.

## 3. Results

### 3.1. Static Balance

#### 3.1.1. CoP_Vm_ Values

In the analysis of the three-way ANOVA, significant main effects were found for Group, Condition, and Surface. As well, significant (Group × Condition), (Surface × Condition), and (Group × Surface) interactions were observed. However, (Group × Surface × Condition) interaction was not statistically significant (Table 2).

On both the firm and foam surfaces, the post hoc analyses revealed that the CoP_Vm_ values significantly increased (*p* < 0.001) in the conditions CE, playing games, watching videos, and video call, compared to the control condition in both the IDG and the TDG (Table 3). However, no significant difference was found between the control condition and the listening to music condition in either the IDG or the TDG (Table 3).

In the IDG, the CoP_Vm_ values significantly increased in the playing game, watching video, and video call conditions (*p* < 0.001, both surfaces), and significantly decreased in the listening to music condition (Firm: *p* = 0.005; Foam: *p* < 0.001) compared to the CE condition. The CoP_Vm_ values significantly increased in the watching videos (Firm: *p* < 0.001; Foam: *p* = 0.003) and video call (*p* < 0.001, only Firm) conditions, and significantly decreased in the listening to music condition (*p* < 0.001, both surfaces) compared to the playing game condition. Moreover, CoP_Vm_ values significantly increased in the video call condition (Firm: *p* < 0.001; Foam: *p* = 0.005), and significantly decreased in the listening to music condition (*p* < 0.001, both surfaces) compared to the watching video condition, as well as significantly decreased in the listening to music condition (*p* < 0.001, both surfaces) compared to the video call condition. However, in the TDG, the CoP_Vm_ values significantly decreased only in the listening to music condition (Firm: *p* = 0.07; Foam: *p* = 0.004; Firm: *p* = 0.03; Foam: *p* = 0.009; Firm: *p* = 0.04; Foam: *p* = 0.01; Firm: *p* = 0.03; Foam: *p* = 0.01) compared to the CE, playing game, watching video, and video call conditions, respectively.

Regarding the effect of Group, the post hoc analyses revealed that the CoP_Vm_ values were significantly lower (*p* < 0.001) in the IDG compared to the TDG in all conditions on both surfaces (Table 3).

Concerning the effect of Surface, the post hoc analyses revealed that the CoP_Vm_ values were significantly greater (*p* < 0.001) on the firm surface compared to the foam surface in all conditions for both the IDG and the TDG.

#### 3.1.2. Δ CoP_Vm_%

For the Δ CoP_Vm_% analysis on the firm surface, the two-way ANOVA revealed significant main effects of Condition and Group. However, there was no significant (Group × Condition) interaction (Table 2). On the foam surface, the main effect of Condition was also significant; however, no significant main effect of Group was observed. In addition, a significant (Group × Condition) interaction was found (Table 2).

In the IDG, the post hoc analyses revealed that the Δ CoP_Vm_% values significantly increased in the playing game, watching video, and video call conditions (*p* < 0.001, both surfaces) and significantly decreased in the listening to music condition (Firm: *p* = 0.016, Foam: *p* < 0.001) compared to the CE condition. Moreover, Δ CoP_Vm_% values decreased in the watching video (Firm: *p* < 0.001, Foam: *p* = 0.025), video call (Firm only: *p* = 0.001), and listening to music (*p* < 0.001, both surfaces) conditions compared to the playing game condition. Moreover, Δ CoP_Vm_% values increased in the video call condition (Firm: *p* = 0.001, Foam: *p* = 0.023) and decreased in the listening to music condition (*p* < 0.001, both surfaces) compared to the watching video condition. Furthermore, Δ CoP_Vm_% values decreased in the listening to music condition (*p* < 0.001, both surfaces) compared to the video call condition (Table 4). However, in the TDG, the results showed that the Δ CoP_Vm_% values significantly increased in the playing game (Firm only, *p* = 0.029) and video call (Firm only, *p* < 0.001) conditions, and decreased in the listening to music condition (*p* < 0.001, both surfaces), compared to the CE condition. In addition, Δ CoP_Vm_% values increased in the video call condition compared to the playing game (Firm only, *p* < 0.001) and watching video (Firm, *p* < 0.001, Foam: *p* = 0.001) conditions. Δ CoP_Vm_% values decreased in the listening to music condition (*p* < 0.001, both surfaces) compared to the playing game and watching video conditions. Furthermore, Δ CoP_Vm_% values decreased in the listening to music condition (*p* < 0.001, both surfaces) compared to the video call condition.

For the effect of Group, the post hoc analyses revealed that the Δ CoP_Vm_% values were significantly greater only on firm surface under the playing game (*p* = 0.011) and video call (*p* = 0.013) conditions (Table 4).

### 3.2. Dynamic Balance

#### 3.2.1. TUGT Scores

In the two-way ANOVA, significant main effects were found for Group and Condition. A significant (Group × Condition) interaction was observed (Table 2).

In the IDG, the post hoc analyses revealed that the TUGT scores significantly increased in the playing game, watching video, and video call conditions (*p* < 0.001), and decreased in the listening to music condition (*p* = 0.005) compared to the control condition. The TUGT scores significantly decreased in the watching video (*p* = 0.017) and listening to music (*p* < 0.001) conditions compared to the playing game condition. Furthermore, the TUGT scores significantly increased in the video call condition (*p* = 0.001) and decreased in the listening to music condition (*p* < 0.001) compared to the watching video condition. The TUGT scores significantly decreased in the listening to music condition (*p* < 0.001) compared to the video call condition. In the TDG, the results showed that the TUGT scores significantly increased in the playing game, watching video, and video call conditions (*p* < 0.001), and decreased in the listening to music condition (*p* = 0.01), compared to the control condition. Moreover, the TUGT scores significantly decreased in the watching video condition (*p* = 0.01) and significantly decreased in the listening to music condition (*p* < 0.001) compared to the playing game condition. The TUGT scores significantly increased in the video call condition (*p* = 0.03) and decreased in the listening to music condition (*p* = 0.049) compared to the watching video condition. The TUGT scores significantly decreased in the listening to music condition (*p* = 0.002) compared to the video call condition.

Regarding the effect of Group, the post hoc analyses revealed that the TUGT scores were significantly lower (*p* < 0.001) in the IDG compared to the TDG in all conditions (Table 3).

#### 3.2.2. Δ TUGT%

In the analysis of Δ TUGT%, significant main effects were found for Group and Condition, as well as a significant (Group × Condition) interaction (Table 2).

In the IDG, the post hoc analyses revealed that the Δ TUGT% significantly decreased in the watching video and listening to music conditions (*p* = 0.003, *p* < 0.001, respectively) compared to the playing game condition. Moreover, the Δ TUGT% significantly increased in the video call condition (*p* < 0.001), and decreased in the listening to music condition (*p* < 0.001) compared to the watching video condition. Furthermore, the Δ TUGT% significantly decreased in the listening to music condition (*p* < 0.001) compared to the video call condition. Regarding the TDG, the results showed that the Δ TUGT% scores significantly decreased in the watching video (*p* = 0.006) and listening to music (*p* = 0.001) conditions compared to the playing game condition. Additionally, the Δ TUGT% significantly increased in the video call condition (*p* = 0.02) and decreased in the listening to music condition (*p* < 0.001) compared to the watching video condition. The Δ TUGT% significantly decreased in the listening to music condition (*p* < 0.001) compared to video call condition (Table 4).

For the effect of Group, the post hoc analyses revealed that the Δ TUGT% were significantly greater in the IDG compared to the TDG in all conditions except the listening to music condition (*p* = 0.04, =0.03, *p* < 0.001, respectively) (Table 4).

## 4. Discussion

Our study aimed to investigate the influence of various smartphone activities, including playing video games, watching videos, engaging in video calls, and listening to music, on the static and dynamic postural balance of adolescents with intellectual disabilities, in comparison with their typically developing counterparts. The main findings revealed that the CoP_Vm_ values and TUGT scores were significantly deteriorated during various smartphone activities (except listening to music) compared to the control condition in both the IDG and the TDG. Our study findings align with previous research in individuals with intellectual disabilities [8,36,37] as well as with typical development [14,38,39] that have shown impaired performance during dual-tasking in various contexts.

### 4.1. Static Postural Balance

Our study showed that CoP_Vm_ values increased during various activities under both surfaces in the IDG and the TDG. Consistent with previous studies [40,41,42,43], smartphone usage was found to have a detrimental effect on posture, particularly in the neck and trunk regions. These alterations in posture could potentially influence the distribution of plantar pressure and affect stabilometric variables [44], as well as increase postural sway and compromised postural control during smartphone activities [43]. Moreover, our findings highlight the importance of postural control in maintaining stability during these tasks. Postural control is a complex process that involves the integration of sensory information from the visual, vestibular, and somatosensory systems to make continuous adjustments and maintain balance [45]. Engaging in activities that challenge postural control can lead to an increased demand on the sensory systems and motor responses involved in maintaining static postural balance. Indeed, playing games on smartphones involves dynamic movements, body position changes, and rapid shifts in attention and focus. These aspects of gaming require continuous adjustments in body posture and weight distribution to maintain stability and adapt to the game’s demands. Consequently, the visual, vestibular, and somatosensory systems, crucial for postural control, must collaborate effectively to provide accurate feedback and facilitate quick motor responses. Furthermore, playing smartphone games engages cognitive processing and motor skills, making it a multifaceted task that presents various challenges to individuals. Previous studies have indicated that cognitive and postural tasks share common cognitive mechanisms, resulting in potential conflicts when both tasks are performed simultaneously. As a result, postural sway has been shown to increase during cognitive tasks in both individuals with intellectual disabilities [26,36,46] and those without [47]. Moreover, watching videos often requires sustained visual attention, and this visual focus can lead to reduced awareness of one’s body position and balance [48]. As individuals become absorbed in the content of the videos, they may unintentionally neglect their postural adjustments, leading to instability and increased postural sway. Engaging in visual tasks, such as video calls or watching videos, can significantly impact postural control, as individuals tend to rely on their visual input to maintain balance. Therefore, the effect of video on postural control can be attributed to the central role of vision in human perception, as the visual system provides important cues for maintaining stability [49]. In addition, during video calls, individuals often maintain a relatively fixed posture while looking at the screen and interacting with others. This sustained static posture, combined with the cognitive demands of engaging in the conversation, can affect postural stability. Our results are in accordance with previous studies in typical developing individuals demonstrating that the ability to maintain static postural balance decreases while talking on the phone [43,50] or engaging in verbal dual-tasking [51]. Furthermore, the increased instability during video calls could be attributed to the demands of articulation, which involve respiratory activity during speech and may contribute to changes in postural sway, as previously demonstrated [43,52,53]. This is not the case for the listening to music condition, as static postural balance was not affected significantly while using the smartphone to listen to music. This was expected, as this condition does not require participants to divide their attention between dual tasks. Listening to music is a common everyday activity for many individuals and is known to have various effects on human physiology and psychology in different populations [33,34,54,55]. However, its influence on postural balance might not always pronounced, especially in the context of static postural balance. It seems that the specific characteristics of the pop music used in this study might not have provided sufficient cognitive or attentional demands to significantly impact postural stability during the static standing task.

In addition, the study results showed that the significant difference in CoP values between the CE condition and engaging in activities like playing video games, watching videos, and video calls in the IDG underscores the unique challenges faced by individuals with intellectual disabilities in maintaining postural balance. The increased postural sway in the IDG participants during these activities indicates that they struggled to maintain a stable posture compared to the CE condition, where sensory inputs are minimized, and they might rely more on proprioceptive and vestibular inputs. Engaging in activities like playing video games and watching videos involves dynamic visual stimuli, which may disrupt postural stability in individuals with intellectual disabilities. Furthermore, video calls may introduce an additional layer of complexity as they require the integration of both auditory and visual inputs, potentially leading to increased postural sway as the IDG participants focus on answering the questions.

It is important to note that, on a firm surface, this study found that the increase in Δ CoP_Vm_% in response to playing games and participating in video calls was more pronounced in the IDG compared to the TDG. This finding suggests that adolescents with intellectual disabilities may have a more significant challenge in maintaining postural stability while performing these activities compared to typically developing adolescents. Indeed, individuals with intellectual disabilities may have differences in sensory processing, which could affect how they perceive and respond to sensory cues related to balance and postural control [3]. It seems that playing games and engaging in video calls, which involve visually complex and dynamic stimuli, as well as multitasking and attention to both visual and auditory stimuli, may pose greater challenges for individuals with intellectual disabilities. These cognitive demands might lead to increased postural sway and difficulties in maintaining static postural balance during these activities. Furthermore, variability in motor skills, attention, and cognitive abilities among participants in the IDG [56] may have contributed to the observed differences in postural sway [3,5,57]. In our study, we observed that participants encountered considerable challenges in dividing their attention between dual tasks during the playing game and video call conditions. These conditions appeared to be more demanding, potentially leading to increased difficulty and inducing fatigue in the postural muscles [58] responsible for maintaining balance.

The analysis of Δ CoP_Vm_% on the foam surface revealed that there were no significant differences in postural sway between the IDG and the TDG across all conditions studied. This finding suggests that the foam surface, which provides an unstable and challenging base of support, had a similar impact on static postural balance for both groups. In fact, the foam surface is known to elicit greater postural instability by reducing the proprioceptive and tactile feedback from the feet [59,60]. This sensory alteration creates a more demanding postural control task for individuals, regardless of their intellectual ability. It seems that both groups may have faced comparable challenges in adapting to the unstable foam surface, resulting in similar postural responses.

### 4.2. Dynamic Balance

Concerning dynamic balance, similar results were observed in the effect of smartphone activities. Playing video games, watching videos, and engaging in video calls led to an increase in TUGT scores in both the IDG and the TDG compared to the control condition. These findings align with previous studies that have reported impaired walking during dual-tasking activities and smartphone use [17,18]. In fact, previous research has demonstrated that engaging in dual-task activities on a smartphone while walking can negatively affect working memory and lead to increased errors, including greater lateral deviations in walking [61]. Another study investigating ground walking mechanics and speed during phone use found that as the complexity of the task increased, the detrimental impact on gait mechanics worsened [62]. These findings highlight the potential risks and challenges associated with using a smartphone while walking, particularly in situations that demand cognitive attention and motor coordination. In our study, it seems that engaging in smartphone activities, such as playing games, watching videos, or participating in video calls, may challenge dynamic balance due to potential divided visual attention and cognitive overload. Participants may have faced difficulties in effectively allocating attention between the video call and the motor task of walking, ultimately affecting their dynamic balance during these activities. Significantly, these results show a more pronounced increase in Δ TUGT% among IDG participants compared to TDG participants. This finding aligns with a previous study involving older adults with Parkinson’s disease, where, when presented with a dual task, they demonstrated a significant reduction in walking speed compared to older adults without Parkinson’s disease [7]. In both cases, the dual-task scenario and the shared impact on dynamic balance underscore the common challenges individuals with specific conditions face when multitasking during walking. In our study, adolescents with intellectual disabilities may experience challenges in attentional control and divided attention [63], making it more difficult for them to allocate their cognitive resources effectively while engaging in such activities, specifically during dynamic balance tasks. This could lead to compromised performance in dynamic balance assessments for the IDG compared to the TDG during these activities.

It is important to note that a unique result emerged in the listening to music condition, where both groups exhibited improved dynamic balance. Participants might have experienced a positive emotional response while listening to music, which could have led to increased confidence and focus [64,65] during the TUGT, contributing to better dynamic balance performance. Music is known to have a profound impact on emotions and mood. In our study we selected pop music with fast tempo. This music genre is widely recognized for its lively and catchy tunes, rhythmic beats, and upbeat melodies [66]. These musical characteristics often evoke positive emotions, energy, and a sense of excitement in listeners [67]. As a result, when individuals are exposed to pop music, they may feel more inclined to move and engage in physical activities [66]. In this context, it seems that the use of pop music with fast tempo could have influenced participants’ movement behaviors during the timed tasks. The energetic and vibrant nature of such genre of music might have inspired participants to adopt a more active and dynamic gait pattern during the walking assessments, such as the TUGT. Importantly, our findings suggest that even brief music exposure can lead to immediate dynamic balance enhancements. Moreover, it has been indicated that music may influence autonomic nervous system activity [68], which could contribute to these acute balance improvements. The influence of different of music genre on postural balance and chronic effects of music should be investigated in future studies.

### 4.3. Strengths and Limitations

This study was the first of its kind to investigate the effects of various smartphone activities on both static and dynamic postural balance in adolescents with intellectual disabilities compared to their typically developing counterparts. The unique focus on smartphone activities provided valuable insights into a relatively unexplored area of research. Moreover, this study assessed a diverse range of smartphone activities, including playing games, watching videos, and engaging in video calls. By examining multiple activities, this study captured a comprehensive understanding of their impact on postural balance. Furthermore, by comparing the results between adolescents with intellectual disabilities and their typically developing counterparts, this study highlighted potential differences in how smartphone activities affect postural balance in these two groups.

Despite the valuable insights gained from our study, several limitations should be acknowledged. While our study’s controlled experimental conditions provided valuable insights, they may not fully capture the complexities of real-world mobile device use, potentially restricting the generalizability of our findings to everyday situations. Future research endeavors should consider conducting investigations in more naturalistic settings, encompassing the dynamic and multifaceted nature of mobile device utilization in daily life, to gain a deeper understanding of its impact on postural balance. In addition, we did not explore the possibility that augmentation of postural sway might, in certain circumstances, represent a positive adaptation to task constraints, affording individuals greater degrees of freedom for dynamic postural adjustments. We also did not employ non-linear methods to investigate the potential benefits of increased noise in postural responses. This limitation underscores the need for future research to delve deeper into these nuances and expand our understanding of postural balance. Moreover, this study primarily focused on static and dynamic postural balance assessments. While these are valuable indicators of postural balance, other factors such as muscle strength and joint stability were not extensively examined. Future research could incorporate a more comprehensive assessment of various aspects of postural balance to gain a greater understanding of the relationship between smartphone activities and postural balance. Likewise, while our study focused on the postural balance task while listening to music, it did not investigate the concept of a “resonant effect” induced by music as well as other variables, such as the type of music, tempo, or individual differences in music preferences, which could have significant effects on postural balance. Future research to include these factors would offer a more comprehensive understanding of the influence of music on postural balance. Furthermore, this study did not investigate the potential long-term effects of smartphone activities on postural balance. This would be essential for considering the broader implications for individuals with intellectual disabilities and typically developing individuals.

### 4.4. Practical Implications

The findings of this study hold significant practical implications for adolescents with intellectual disabilities, healthcare professionals, and caregivers alike. In fact, educating caregivers about the potential effects of smartphone activities on postural balance is crucial. By raising awareness and providing guidance on responsible smartphone use, caregivers can play a vital role in ensuring the safety and well-being of adolescents with intellectual disabilities. In addition, collaboration between healthcare professionals, educators, therapists, and technology experts can lead to the development of innovative solutions and interventions to enhance postural balance during smartphone activities. Long-term monitoring is also recommended to track changes in postural control and balance over time. By regularly assessing postural balance, healthcare professionals can identify trends and implement timely interventions, contributing to better outcomes for individuals with intellectual disabilities. These practical measures align with the broader goal of preventing and reducing the risk of falls, ultimately enhancing the quality of life for adolescents with intellectual disabilities.

## 5. Conclusions

This study highlighted that engaging in smartphone activities, including playing video games, watching videos, and participating in video calls, has significant effects on both static and dynamic postural balance in both groups. These activities present challenges to postural balance and can lead to increased postural sway, affecting the performance during tasks like the TUGT. Moreover, adolescents with intellectual disabilities demonstrated more pronounced alterations in postural balance in response to playing games and video call smartphone activities compared to their typically developing counterparts. This highlighted the unique challenges that smartphone interactions pose for adolescents with intellectual disabilities, with cognitive and attentional demands potentially playing a crucial role in affecting their postural balance during these tasks. In addition, the finding that listening to music led to increased dynamic balance scores in both groups raises interesting questions about the influence of music genre on postural responses. Future research could explore the specific effects of different music genres on postural control to gain a deeper understanding of this phenomenon. In practical term, our study highlights the need for assistive devices and caregiver education to improve smartphone usage safety for adolescents with intellectual disabilities. Collaboration between healthcare professionals, educators, therapists, and technology experts can lead to the development of innovative solutions and interventions to enhance postural balance during smartphone activities in order to prevent and reduce the risk of falls, ultimately enhancing individuals’ quality of life.

## Figures and Tables

**Table 1:** Participants’ characteristics.

	IDG (*n* = 16)		TDG (*n* = 12)		Degree of Freedom	Independent *t*-Test
	Mean ± SD	Coefficient of Variation	Mean ± SD	Coefficient of Variation		
Age (years)	14.50 ± 1.15	7.93	14.75 ± 1.05	7.12	26	*p* = 0.55
Height (cm)	152.83 ± 3.78	2.47	152.75 ± 4.11	2.69	26	*p* = 0.43
Mass (kg)	46.25 ± 4.11	8.88	47.76 ± 3.16	6.63	26	*p* = 0.43
BMI	19.78 ± 1.38	6.97	20.49 ± 1.50	7.32	26	*p* = 0.58

**Table 2:** Summary of ANOVA results.

	F	Degree of Freedom	*p*	ηp^2^
CoP_Vm_				
Group	122.36	1.26	<0.001	0.81
Condition	48.49	5.22	<0.001	0.89
Group × Condition	19.39	5.22	<0.001	0.64
Surface	0.86	1.26	<0.001	0.88
Group × Surface	3.28	1.26	=0.001	0.35
Surface × Condition	6.40	5.22	=0.001	0.59
Group × Surface × Condition	1.55	5.22	=0.21	-
Δ CoP_Vm_%				
Firm surface				
Group	5.42	1.26	=0.028	0.20
Condition	52.07	4.23	<0.001	0.90
Group × Condition	1.76	4.23	=0.17	-
Foam surface				
Group	0.48	1.26	0.49	-
Condition	53.83	4.23	<0.001	0.90
Group × Condition	3.25	4.23	=0.030	0.36
TUGT				
Group	82.84	1.26	<0.001	0.76
Condition	65.75	4.23	<0.001	0.90
Group × Condition	7.45	4.23	=0.001	0.56
Δ TUGT%				
Group	13.06	1.26	=0.001	0.34
Condition	212.65	3.24	<0.001	0.91
Group × Condition	5.77	3.24	=0.004	0.41

**Table 3:** Means ± SD of mean velocity CoP (CoP_Vm_) values and Timed Up-and-Go Test (TUGT) scores for control, closed eyes (CE), playing games, watching video, video calls, and listening to music conditions on firm and foam surfaces in the intellectual disability group (IDG) and the typical developing group (TDG).

	IDG	TDG	Control vs. Conditions IDG		Control vs. Conditions TDG		IDG vs. TDG	
	Means ± SD (95% CI)	Means ± SD (95% CI)	*p*-Value	d	*p*-Value	d	*p*-Value	d
CoP_Vm_								
Firm control	13.10 ± 2.83 (11.59 to 14.61)	7.14 ± 1.83 (5.99 to 8.30)	-	-	-	-	<0.001	2.50
Firm CE	16.69 ± 2.66 (15.27 to 18.11)	9.29 ± 1.72 (8.14 to 10.39)	<0.001	1.30	<0.001	1.22	<0.001	3.30
Firm playing game	26.31 ± 6.21 * (22.99 to 29.62)	11.16 ± 1.31 (10.32 to 12.00)	<0.001	2.73	=0.03	2.52	<0.001	3.37
Firm watching video	17.83 ± 3.85 *$ (11.70 to 16.59)	9.58 ± 1.75 (8.47 to 10.69)	<0.001	1.39	=0.01	1.36	<0.001	2.75
Firm video call	30.77 ± 8.04 *$£ (26.48 to 35.06)	12.58 ± 1.55 (11.59 to 13.57)	<0.001	2.93	=0.04	3.20	<0.001	3.14
Firm listening to music	13.85 ± 4.22 *$£# (11.60 to 16.11)	6.19 ± 1.34 *$£# (5.34 to 7.04)	=0.88	-	=0.85	-	<0.001	2.44
Foam control	20.59 ± 4.31 (18.30 to 22.89)	11.25 ± 2.82 (9.45 to 13.05)	-	-	-	-	<0.001	2.55
Foam CE	27.15 ± 6.70 (23.57 to 30.72)	16.79 ± 2.55 (14.71 to 17.96)	<0.001	1.16	=0.004	2.06	<0.001	2.04
Foam playing game	41.56 ± 7.88 * (37.36 to 45.76)	18.15 ± 3.52 (15.92 to 20.39)	<0.001	3.30	=0.040	2.16	<0.001	3.83
Foam watching video	35.08 ± 8.16 *$ (30.72 to 39.43)	17.49 ± 2.59 (15.85 to 19.14)	<0.001	2.22	=0.046	2.30	<0.001	2.90
Foam video call	42.77 ± 7.22 *£ (38.82 to 46.62)	20.41 ± 5.11 (17.16 to 23.66)	<0.001	3.73	=0.006	2.21	<0.001	3.57
Foam listening to music	18.56 ± 3.79 *$£# (16.54 to 20.59)	10.00 ± 2.26 *$£# (8.56 to 11.44)	=0.13	-	=0.80	-	<0.001	2.74
TUGT								
Control	10.53 ± 1.03 (9.98 to 11.07)	7.88 ± 0.79 (7.38 to 8.38)	-	-	-	-	<0.001	2.88
Playing game	19.55 ± 3.77 (17.54 to 21.56)	12.70 ± 1.94 (11.47 to 13.94)	<0.001	3.27	<0.001	3.26	<0.001	2.28
Watching video	15.25 ± 4.68 $ (12.76 to 17.74)	9.57 ± 1.11 $ (8.86 to 10.28)	<0.001	1.39	<0.001	1.75	<0.001	1.66
Video call	21.47 ± 4.59 $£ (19.01 to 23.90)	11.57 ± 0.71 £ (11.11 to 12.02)	<0.001	3.28	=0.01	2.62	<0.001	2.99
Listening to music	9.91 ± 0.91 $£# (9.42 to 10.40)	7.09 ± 0.55 $£# (6.79 to 7.42)	=0.005	0.63	=0.002	1.16	<0.001	2.71

Notes: *: Significant difference (*p* < 0.05) between the closed eyes (CE) and the playing games, watching videos, video call, and listening to music conditions. $: Significant difference (*p* < 0.05) between the playing game and the watching video, video call, and listening to music conditions. £: Significant difference (*p* < 0.05) between the watching videos and the video call and listening to music conditions. **#**: Significant difference (*p* < 0.05) between the video call and listening to music conditions.

**Table 4:** Means ± SD of Δ changes of mean velocity CoP values (Δ CoP_Vm_%) and Timed Up-and-Go Test scores (Δ TUGT%) for control, closed eyes (CE), playing game, watching video, video call, and listening to music conditions on firm and foam surfaces in the intellectual disability group (IDG) and the typical developing group (TDG).

	IDG	TDG	IDG vs. TDG	
	Means ± SD (95% CI)	Means ± SD (95% CI)	*p*-Value	d
Δ CoP_Vm_%				
Firm CE	21.75 ± 8.52 (17.21.59 to 26.30)	22.57 ± 16.70 (11.95 to 33.18)	=0.086	-
Firm playing game	48.79 ± 11.40 * (42.71 to 54.87)	36.13 ± 13.10 * (27.80 to 44.48)	=0.011	1.03
Firm watching video	33.53 ± 10.87 *$ (27.72 to 39.34)	25.61 ± 11.13 (18.53 to 32.69)	=0.073	-
Firm video call	55.46 ± 11.46 *$£ (49.35 to 61.57)	43.02 ± 13.17 *$£ (34.64 to 51.39)	=0.013	1.01
Firm listening to music	−0.99 ± 32.71 *$£# (−18.42 to 16.43)	−15.10 ± 10.03 *$£# (−21.48 to −8.72)	=0.162	-
Foam CE	22.18 ± 14.43 (14.48 to 29.87)	31.64 ± 8.68 (26.12 to 37.15)	=0.050	-
Foam playing game	48.56 ± 15.80 * (40.13 to 56.98)	36.76 ± 16.14 (26.50 to 47.01)	=0.060	-
Foam watching video	38.05 ± 19.65 *$ (27.57 to 48.52)	35.99 ± 9.91 (29.69 to 42.29)	=0.074	-
Foam video call	50.26 ± 15.18 *£ (42.17 to 58.35)	42.64 ± 17.15 £ (31.63 to 53.43)	=0.21	-
Foam listening to music	−11.52 ± 12.68 *$£# (−18.28 to −4.72)	−16.63 ± 34.81 *$£# (−38.81 to 5.48)	=0.59	-
Δ TUGT%				
Playing game	44.54 ± 10.19 (39.10 to 49.37)	36.23 ± 9.44 (27.68 to 44.77)	=0.04	0.81
Watching video	26.70 ± 10.91 $ (26.62 to 34.61)	17.30 ± 6.88 $ (12.92 to 21.67)	=0.03	1.03
Video call	48.93 ± 11.34 £ (42.88 to 54.97)	30.31 ± 13.06 £ (22.00 to 38.60)	<0.001	2.01
Listening to music	−6.25 ± 3.53 $£# (−8.13 to −4.36)	−11.54 ± 13.12 £$# (−19.88 to −3.20)	=0.13	-

Notes: *: Significant difference (*p* < 0.05) between the closed eyes (CE) and the playing game, watching video, video call, and listening to music conditions. $: Significant difference (*p* < 0.05) between the playing game and the watching video, video call, and listening to music conditions. £: Significant difference (*p* < 0.05) between the watching videos and the video call and listening to music conditions. **#**: Significant difference (*p* < 0.05) between the video call and listening to music conditions.

## Data Availability

The datasets used and/or analyzed during the current study are available from the corresponding author upon reasonable request.
